# Supplementary material for: HBV-RNA, Quantitative HBsAg, Levels of HBV in Peripheral Lymphocytes and HBV Mutation Profiles in Chronic Hepatitis B
Source: Viruses. 2022 Mar 11;14(3):584. doi: 10.3390/v14030584 (PMC8949614; doi:10.3390/v14030584)
Supplement: Supplementary file 1 [file viruses-14-00584-s001.zip › viruses-1592636-supplementary.pdf]

Supplementary Materials:

Table S1: HBV mutations by genotype and HBeAg status

Type of mutations	Position (amino or nucleic acids)*	Genotype			HBeAg	
		A	C	D	Positive	Negative
Resistance	204I			1	1	
	173L, 180M and 204V			1		1
Escape**	120P/T/S	1***		1	1	1
	122K			2	1	1
	126N/I		1	1		2
	128V			4		4
	131N			2		2
	132F			1		1
	133T			1		1
	134H/N			2	1	1
	143L			1		1
	145R			1	1	
BCP	A1762T			3		3
	G1764A			7		7
	A1762T+ G1764A	3	1	16	1	19
	no mutation	1		30	3	28
	Total	4	1	56	4	57
PC***	G1896A			6	1	5
	G1899A		1	21		22
	G1896A+ G1899A			24		24
	no mutation	4		6	3	7
	Total	4	1	57	4	58

*Position of resistance and escape mutations is in amino-acids and BCP/PC mutations are according to the nucleic acid position. **Five patients had more than one mutation. ***One patient had both 120P and 120T mutation. ***Three of the six patients without any G1896A or G1899A mutations, had mutations in the initiation codon: two with genotype D had G1816T mutation and one with genotype A had T1815C mutation.
